# Pennsylvania State Dental Society

**Published:** 1885-09

**Authors:** William H. Trueman

**Affiliations:** Philadelphia


### PENNSYLVANIA STATE DENTAL SOCIETY.

### SEVENTEENTH ANNUAL SESSION, HELD AT CRESSON, JULY 28, 29, AND 30, 1885.

REPORTED FOR THE "INDEPENDENT PRACTITIONER" BY WILLIAM H. TRUEMAN, PHILADELPHIA.

### TUESDAY, JULY 28—MORNING SESSION.

The meeting was called to order at eleven o'clock by the President, Dr. George Elliott, of Meadville. About forty members and delegates were present.

The morning session was taken up with routine business, the only matter of general interest being the reading of a communication received from a committee consisting of Drs. T. W. Brophy, C. R. E. Koch, and J. N. Crouse, appointed by the Chicago Dental Society, at a meeting held Dec., 1884, to take action in reference to the meeting of the International Medical Congress at Washington in 1887. The communication requested all who were in favor of organizing a section of Oral and Dental Surgery in the International Medical Congress to use their influence to have it so organized, and that all practitioners of Dentistry, recognized by Dental Societies, may be eligible to membership or admission to the Congress.

On motion, the communication was referred to a committee consisting of Drs. Louis Jack, C. N. Pierce and E. T. Darby.

Adjourned.

At the opening of the afternoon session the above committee made the following report, which was unanimously adopted :

*To the Pennsylvania State Dental Society :*

Your committee, to whom was referred the communication of Drs. Brophy, Koch, and Crouse, of the Chicago Dental Society, would recommend this Society to send to the American Dental Association, at the meeting of this year, the following preamble and resolution :

PREAMBLE, This Society recognizes with satisfaction that the International Medical Congress has in its organization included a section of Oral and Dental Surgery.

*Resolved,* That it is the sentiment of this Society, that the organization and personnel of the section of Oral and Dental Surgery should be instituted by the American Dental Association, which we believe in its corporate capacity is the only body fitted to satisfactorily establish said section.

We also recommend the Society to communicate with the officers of the International Medical Congress, and to suggest the propriety of placing the appointment of the members of the section of Oral and Dental Surgery, and the organization of this section, in the power of the American Dental Association; and that the Society takes this action in the belief that the result of such action by a representative association, composed of the leading persons in this country engaged in the study and practice of Oral and Dental Science, will prove entirely satisfactory.*

Signed, LOUIS JACK, *Chairman.*

EDWIN T. DARBY,

C. N. PIERCE.

An address of welcome was then read by Dr. W. B. Miller, of Altoona, and responded to on behalf of the society by Dr. Gerhart, of Lewisburg. This was followed by the President's address, all of which, while quite appropriate, had no special professional bearing.

The meeting being open for professional topics, Dr. C. N. Pierce asked if it was possible for a patient to be so susceptible to the influence of mercury that an amalgam filling in the crown of a vital molar would cause pericemental inflammation.

He related the case of a lady who came to him complaining of discomfort in two adjoining molar teeth; the teeth were rather loose, quite sore, and gave every evidence of marked pericemental inflammation. On two occasions, while under medical treatment, the patient had suffered ptyalism from minute doses of some mercurial preparation, the effect being induced on each occasion accidentally, and much to the physician's surprise, thus showing a marked susceptibility to the drug. The fillings were removed and gutta-percha substituted, with almost immediate relief. In a short time the soreness disappeared, and the teeth became quite firm. The patient then called attention to two molar teeth on the other side of the mouth, also filled with amalgam. They were uncomfortable, but not so painful as those first treated. The amalgam was removed, the teeth giving every evidence of vitality. They were refilled with gutta-percha and became quite comfortable and

* Readers of the INDEPENDENT PRACTITIONER are aware that the reorganized committee of the American Medical Association has now dropped the section of Dental and Oral Surgery entirely, but this report of the committee of the Pennsylvania Society is published as indicative of the feeling of representative men of the profession.—EDITOR.

firm. The case had been one of much interest to him; there had been no treatment farther than substituting the gutta-percha for the amalgam. A similar case had been sent to him by a dentist, who was treating an apparently vital tooth for pericemental inflammation without satisfactory results. This also contained a large amalgam filling. He suggested that it should be removed and replaced with gutta-percha, which was done with entire relief. In each case cited the fillings were confined to the crowns, were in excellent condition, and were not in contact with the soft tissues at any point; the effect seemed to be purely local, the only evidence of the action of the mercury being the condition of the teeth, which were in each case sore, loose, and elongated.

*Dr. E. T. Darby*—Stated that about twenty years ago, while visiting a dental office in the western part of New York, he was shown a number of teeth markedly exostosed, most of them containing amalgam fillings. They had been recently extracted from a lady's mouth. The patient had complained of severe neuralgic pain, and after suffering for some time the teeth were extracted. Those containing the amalgam fillings were markedly exostosed, while the others appeared to be normal, and if he remembered right, were sound and healthy. The dentist who extracted them thought that the exostosis was in some way due to the amalgam. The incident was recently recalled by receiving from a dentist residing in the West a box containing a large number of specimens of exostosed teeth. On examining these he was surprised to find that a large proportion contained amalgam fillings. He was not prepared to say how the amalgam caused the exostosis, nor yet even to say that it did do so. The remarks of Dr. Pierce reminded him of it.

The discussion became general on the demerits and merits of amalgam, the question of Dr. Pierce being lost sight of.

*Dr. G. L. Robb*—Said that some time ago he was annoyed by the large number of teeth he filled with amalgam coming back to him, the patients complaining of severe pain, generally of a darting character, as though the pulp was pressed upon. He at last thought it might be due to the expansion of the amalgam. He discarded the make he was then using, and began using another, since which time he has had no trouble.

*Dr. C. S. Beck*—Had a case very much like that related by Dr. Pierce. The patient was a lady of an excitably nervous disposition.

Some time after an amalgam filling had been inserted, the tooth became troublesome. He removed the amalgam and replaced it with gutta-percha, since when it has been quite comfortable. He could assign no reason for the trouble.

*Dr. William H. Trueman*—Had listened with interest to the remarks of Drs. Pierce and Darby. He could not conceive that so small an amount of mercury as would escape from an amalgam filling could cause the severe local trouble spoken of without its presence being made known in other ways; and yet, we must bear in mind that there are individuals remarkably susceptible, not only to some particular drug, but also to some one marked therapeutic property of that drug. If the teeth spoken of should, after a time, be again filled with amalgam, and the symptoms previously observed recur, that would be very strong evidence that the mercury was the cause. He had met with a number of cases where pain followed the filling of a large cavity (in a vital tooth) with amalgam, and which were relieved by removing the filling and substituting gutta-percha, or one of the cements; these same teeth, after a time, were again filled with amalgam and remained comfortable. The pain complained of was more a soreness than that which usually arises from thermal changes. In these cases the amalgam evidently was not the real cause of the trouble.

With regard to the teeth spoken of by Dr. Darby, we must not forget the original lesion, the cavity of decay, which preceded the amalgam filling, and which of itself would be sufficient cause for the condition noted. We all know how slight a cause will occasionally produce exostoses, and how frequently that deviation from the normal condition is met with, and how frequently we meet with marked cases of it where there is no apparent cause of any kind, and where otherwise the teeth and their surroundings appear to be sound and healthy. If we should meet with a mouth containing some teeth filled with gold or gutta-percha, and some with amalgam, and on extraction find those filled with amalgam exostosed while the others were not, we might safely consider that as conclusive evidence that the amalgam had something to do with it. While it will not do to entirely ignore these mysterious cases, it is equally unwise to give to them undue importance. They are entitled to careful consideration and study. He was reminded by the remarks made of discussions upon the same subject which took place forty or fifty years ago. Men who stood quite as well with the profession then as we do now related cases where they had inserted amalgam fillings in teeth that had been dead for years—without any previous treatment—and when the inevitable alveolar abscess made its appearance it was spoken of and believed to be due entirely to the poisonous effect of the amalgam. These cases were constantly cited as reasons why amalgam should never be used in the mouth. Those who spoke then undoubtedly gave utterance to their honest convictions, and believed every word they said; in the light of the present, we are amused at their blunders. This should teach us caution.

Adjourned.

(TO BE CONTINUED.)

